# The effect of continuous glucose monitoring on neonatal outcomes in pregnant women with diabetes

**DOI:** 10.3389/fendo.2026.1815133

**Published:** 2026-03-25

**Authors:** Xiaojun Lai, Jiayi Yu, Shutao Zhu, Kai Zhang, Xiabin Ye

**Affiliations:** 1 Department of Obstetrics and Gynecology, The First Affiliated Hospital of Lishui University, Lishui People’s Hospital, Lishui, China; 2 Department of Critical Care Medicine, Second Affiliated Hospital of Zhejiang University School of Medicine, Hangzhou, China

**Keywords:** continuous glucose monitoring, gestational diabetes mellitus, meta−analysis, neonatal outcomes, systematic review

## Abstract

**Objective:**

To evaluate the effects of continuous glucose monitoring (CGM) compared with standard self−monitoring of blood glucose (SMBG) on neonatal perinatal outcomes among pregnant women with diabetes.

**Methods:**

A systematic search of PubMed, Embase, Scopus, Cochrane library was conducted from database inception through February 5th, 2026. Randomized controlled trials (RCTs) enrolling pregnant women with confirmed diabetes that compared CGM with conventional SMBG were included. Primary outcomes included large for gestational age (LGA), small for gestational age (SGA), neonatal hypoglycemia, neonatal hyperbilirubinemia, and admission to the neonatal intensive care unit (NICU). Random−effects meta−analyses were performed to calculate pooled risk ratios (RRs) with 95% confidence intervals (CIs).

**Results:**

Seventeen RCTs comprising 2,349 women with diabetes were included, with 1,234 participants allocated to CGM and 1,115 to SMBG. CGM use was associated with a significantly lower risk of NICU admission compared with SMBG (RR 0.75, 95% CI 0.59 to 0.97, P = 0.03, I2 = 0%). CGM demonstrated a nonsignificant trend toward reduced incidence of LGA (RR 0.81, 95% CI 0.65 to 1.01, P = 0.06, I2 = 31%) and neonatal hypoglycemia (RR 0.85, 95% CI 0.70 to 1.03, P = 0.10, I2 = 0%). However, CGM was associated with an increased risk of SGA (RR 1.44, 95% CI 1.01 to 2.04, P = 0.04, I2 = 0%). No significant difference was observed in the risk of neonatal hyperbilirubinemia between groups (RR 0.81, 95% CI 0.65 to 1.01, P = 0.64, I2 = 0%). Between−study heterogeneity was low to moderate across outcomes.

**Conclusion:**

Among women with diabetes, CGM is associated with a significant reduction in NICU admissions. CGM may reduce the risk of LGA and neonatal hypoglycemia, although the beneficial effects did not reach statistical significance. The observed increase in SGA highlights the need for careful glycemic target optimization when using CGM in GDM. Further large, high−quality RCTs are warranted to define optimal CGM−guided glucose targets that balance fetal overgrowth and growth restriction.

## Introduction

Diabetes in pregnancy, which includes both pregestational diabetes (type 1 and type 2 diabetes) and gestational diabetes mellitus (GDM), affects over 21 million live births annually worldwide, accounting for approximately 17% of all live births (1, 2). Diabetes in pregnancy is associated with increased risks of adverse maternal and neonatal outcomes, including preeclampsia, cesarean delivery, large for gestational age (LGA) infants, neonatal hypoglycemia, and neonatal intensive care unit (NICU) admission (3, 4). Achieving optimal maternal glycemic control is a central goal of GDM management, as maternal hyperglycemia is a key modifiable determinant of these complications (5). Optimal glycemic control is central to reducing these risks, and current management strategies rely primarily on dietary intervention, pharmacotherapy when indicated, and self−monitoring of blood glucose (SMBG) (6).

Continuous glucose monitoring (CGM) provides near−real−time interstitial glucose measurements and enables assessment of glycemic variability and time−in−range, offering important advantages over traditional SMBG (7). In pregnant women with diabetes, accumulating evidence from randomized controlled trials (RCTs) and systematic reviews has consistently demonstrated that CGM use is associated with improved maternal glycemic outcomes, including lower mean glucose levels, reduced glycemic variability, and increased time−in−range (8, 9). Despite evidence supporting CGM for optimizing maternal glucose control in pregnancy, its effect on neonatal outcomes is unclear. Although CGM may facilitate earlier detection of postprandial hyperglycemia and nocturnal dysglycemia, RCTs evaluating CGM in women with GDM have reported inconsistent effects on clinically important neonatal outcomes. Individual trials have variably shown reductions, no differences, or even increases in LGA, small for gestational age (SGA), neonatal hypoglycemia, and cesarean delivery (10–12). Moreover, study designs have differed substantially with respect to CGM device type, duration of use (intermittent vs continuous), glycemic targets, and concomitant treatment algorithms, complicating interpretation and limiting generalizability.

Several systematic reviews and meta−analyses have attempted to synthesize the available evidence on CGM use in neonatal outcomes (8, 9, 13, 14). While some have suggested modest improvements in neonatal birth weight (13, 14), most pooled analyses included small trials with limited power, combined women with gestational and pregestational diabetes, or incorporated nonrandomized studies, thereby weakening conclusions regarding neonatal outcomes. In addition, emerging trial data raise concern that intensified glucose monitoring and tighter glycemic targets may inadvertently increase the risk of fetal growth restriction, highlighting the possibility of unintended harm with universal CGM adoption in GDM (10, 15).

Given the rapidly increasing clinical use of CGM in pregnancies complicated by diabetes despite ongoing uncertainty regarding its benefits and risks across different diabetes phenotypes, a comprehensive and methodologically rigorous synthesis of RCTs is urgently needed. Therefore, we conducted a systematic review and meta−analysis of randomized controlled trials comparing CGM with SMBG in pregnant women with diabetes, including type 1 diabetes (T1D), type 2 diabetes (T2D), and GDM, with the aim of evaluating clinically relevant neonatal outcomes and exploring potential effect modification by diabetes type and CGM modality.

## Methods

### Protocol registration and reporting standards

This systematic review and meta−analysis adhered to the Preferred Reporting Items for Systematic Reviews and Meta−Analyses (PRISMA) 2020 statement (16) (the checklist is available in **Supplementary Material 1**). The study protocol was prospectively registered on the Open Science Framework platform (https://osf.io/ubc68).

### Data sources and search strategy

A comprehensive literature search was conducted in PubMed, Embase, Scopus, and the Cochrane Library from database inception through February 5th, 2026. The search strategy integrated medical subject headings (MeSH) and free-text terms related to diabetes and glucose monitoring. Key search terms included “diabetes”, “continuous glucose monitoring”, “pregnancy”, and “randomized controlled trial”. The complete search strategy for each database is provided in **Supplementary Table 1**. Additionally, reference lists of all included studies and relevant systematic reviews were manually screened to identify additional eligible trials.

### Study eligibility criteria

Studies were included if they met the following criteria:

Population: Pregnant women with a confirmed diagnosis of T1D, type T2D, or GDM, defined according to recognized international or national diagnostic criteria.

Intervention: Use of CGM, including real−time or intermittently scanned CGM, for any duration during pregnancy.

Comparator: Standard SMBG using capillary finger−stick testing.

Outcomes: Reporting at least one of the predefined neonatal outcomes of interest, including LGA, SGA, neonatal hypoglycemia, neonatal hyperbilirubinemia, admission to the neonatal intensive care unit (NICU). Definitions of outcomes were accepted as reported in the original trials. When multiple definitions were used, priority was given to clinically standard definitions (e.g., LGA or SGA defined as birth weight > 90th or < 10th percentile for gestational age).

Study design: RCTs published in English.

Studies were excluded if they included women without separate GDM−specific data, were observational, quasi−experimental, crossover without parallel control, or nonrandomized studies, used CGM exclusively in the intrapartum or immediate postpartum period, did not include a SMBG comparator group, were available only as abstracts, conference proceedings, or unpublished data.

### Study selection and data extraction

All records identified through the search were imported into reference management software, and duplicates were removed. Two reviewers independently screened titles and abstracts for eligibility. Full texts of potentially relevant studies were then assessed independently for inclusion. Discrepancies at any stage were resolved through discussion or consultation with a third reviewer. The study selection process is summarized in a PRISMA flow diagram.

Data were extracted independently by two reviewers using a standardized, pilot-tested data extraction form. Extracted information included study characteristics (e.g., author, year, country, diabetes types, sample size), intervention details, and neonatal outcomes. When outcome data were incomplete or unclear, the original authors were contacted for clarification where feasible.

### Risk of bias assessment

The risk of bias for each included RCT was assessed independently by two reviewers using the Cochrane Risk of Bias 2.0 tool (17). Specific domains evaluated included the randomization process, deviations from intended interventions, missing outcome data, outcome measurement, and selection of reported results. Each domain was rated as low risk, some concerns, or high risk, and an overall risk-of-bias judgment was assigned per study. Disagreements were resolved through consensus.

### Data synthesis and statistical analysis

Meta−analyses were performed using a random−effects model (DerSimonian and Laird method) to account for anticipated clinical and methodological heterogeneity across studies. For dichotomous outcomes, pooled risk ratios (RRs) with 95% confidence intervals (CIs) were calculated. Statistical heterogeneity was assessed using the Cochran Q test and quantified with the I² statistic, with values of 25%, 50%, and 75% representing low, moderate, and high heterogeneity, respectively (18).

Prespecified subgroup or sensitivity analyses were conducted when data permitted, including analyses based on type of CGM (real−time [RT], intermittently scanned), types of diabetes (T1D, T2D, or GDM). Sensitivity analysis via iterative single-study omission was undertaken to evaluate finding robustness.

Dissemination bias was evaluated through Egger’s parametric test (restricted to outcomes with ≥ 10 studies), and graphical examination of funnel plot symmetry (19). Where funnel asymmetry was indicative of small-study effects, trim-and-fill adjustment was performed to evaluate estimate durability under alternative bias assumptions (20). Conflicting assessments across all review phases were harmonized through iterative team consensus.

All statistical analyses were performed using R statistical software (“meta” and “robvis” package) (21, 22). A two−sided P value <0.05 was considered statistically significant.

## Results

### Study selection and study characteristics

**Figure 1:**
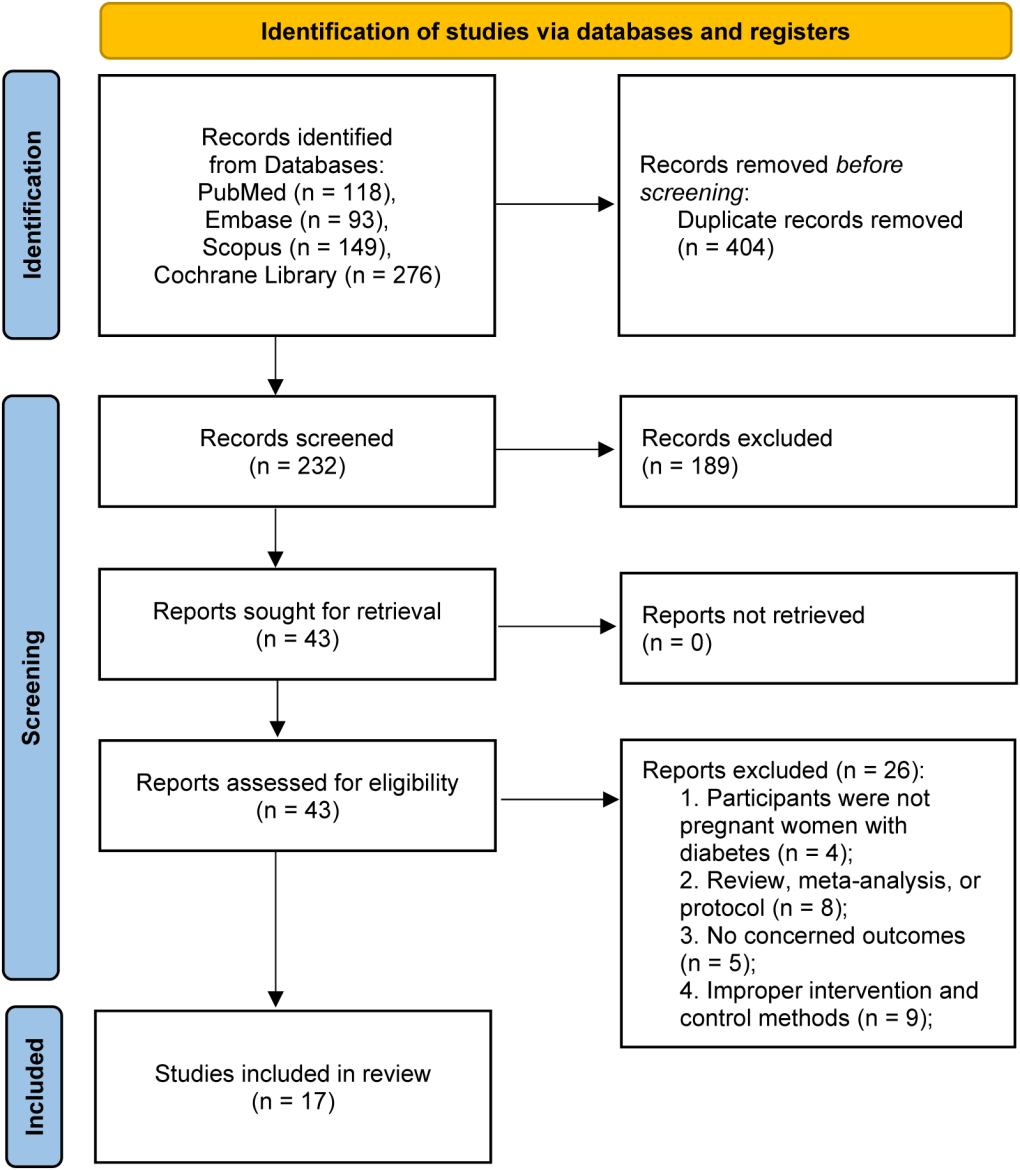
PRISMA 2020 flow diagram for this systematic review and meta-analysis.

The study selection process is detailed in the PRISMA flow diagram (**Figure 1**). A total of 636 records were initially identified through systematic searches of four databases: PubMed (n=118), Embase (n=93), Scopus (n=149), and the Cochrane Library (n=276). After removing 404 duplicate records, 232 unique records were screened based on their titles and abstracts. Of these, 189 records were excluded, leaving 43 reports sought for full-text retrieval. All 43 full-text articles were successfully retrieved and assessed for eligibility. During the full-text review, 26 articles were excluded for various reasons. Consequently, 17 studies met all pre-specified eligibility criteria and were included in the final systematic review and meta-analysis (10–12, 15, 23–35) (**Figure 1**).

**Table 1** summarized the characteristics of the included studies. The 17 included RCTs comprised a total of 2,349 pregnant women with diabetes, of whom 1,234 were assigned to CGM and 1,115 to SMBG. Publication years ranged from 2007 to 2026, and studies were conducted across diverse geographic settings including Europe, North America, and Asia. Most studies focused on GDM, whereas the remaining trials enrolled women with T1D and/or T2D, either exclusively or in mixed diabetes populations. Regarding intervention modality, real-time CGM was the most frequently used approach (10/17 trials), followed by retrospective CGM (5/17 trials) and intermittently scanned CGM (2/17 trials). Regarding CGM technology, the included studies employed various devices across different generations. Real-time CGM systems included Dexcom G6 Pro in 1 study, Dexcom G6 in 4 studies, Medtronic iPro 2 in 3 studies, and Medtronic Guardian REAL-Time in 2 studies. Intermittently scanned CGM was represented by the FreeStyle Libre system (Abbott) in 2 studies. Retrospective CGM using Medtronic systems was employed in 5 studies (Medtronic iPro 2 in 2 studies, MiniMed Gold in 3 studies). The evolution from earlier-generation devices to more recent systems with improved accuracy, user interface, and data-sharing capabilities reflects the rapid technological advancement in CGM during the study period.

**Table 1:** Characteristics of included studies.

Study, year, country	Types of diabetes	Sample size (CGM/SMBG)	Intervention, CGM Device	Glucose target
GRACE trial 2026, Austria, Switzerland, Germany	GDM	190/185	Real-time CGM (Dexcom G6 Pro); SMBG for 4 times/day	Fasting glucose ≤ 95 mg/dL, 1 h postprandial glucose ≤ 140 mg/dL
Ehrhardt 2026, USA	GDM	46/21	Real-time CGM (Dexcom G6); SMBG for 4 times/day	Fasting glucose ≤ 90 mg/dL, 1 h postprandial glucose ≤ 140 mg/dL
Elkind-Hirsch 2025, USA	GDM	80/40	Real-time CGM (Dexcom G6); SMBG for 4 times/day	Fasting glucose ≤ 90 mg/dL and 1 h postprandial glucose ≤ 120 mg/dL
DipGluMo trial 2025, Switzerland	GDM	156/143	Real-time CGM (Dexcom G6); SMBG for 6 times/day	Fasting glucose ≤ 5.3 mmol/L, 1 h postprandial glucose ≤ 8 mmol/L
Valent 2025, USA	GDM	74/37	Real-time CGM (Dexcom G6); SMBG for 4 times/day	Fasting glucose ≤ 95 mg/dL, 1 h postprandial glucose ≤ 140 mg/dL
Lai 2023, China	GDM	62/62	Real-time CGM (Medtronic iPro 2, Medtronic); SMBG for 4 times/day	Fasting glucose ≤ 5.3 mmol/L, 1 h postprandial glucose ≤ 7.8 mmol/L
FLAMINGO trial 2023, Poland	GDM	49/50	Intermittently scanned CGM (Freestyle Libre, Abbott); SMBG for 4 times/day	Fasting glucose ≤ 90 mg/dL and 1 h postprandial glucose ≤ 140 mg/dL
Tumminia 2021, Italy	T1D, T2D	21/19	Intermittently scanned CGM (Freestyle Libre, Abbott); SMBG for 6 times/day	NR
Lane 2019, USA	GDM	12/11	Real-time CGM (Medtronic iPro 2, Medtronic); SMBG for 4 times/day	Glucose 70 to 140 mg/dL
Paramasivam 2018, Malaysia	GDM	25/25	Retrospective CGM (Medtronic iPro 2, Medtronic); SMBG for 4 times/day	Fasting glucose ≤ 5 mmol/L, 2 h postprandial glucose ≤ 6.7 mmol/L
GlucoMOMS trial 2018, Netherlands, Belgium	T1D, T2D, GDM	147/153	Retrospective CGM (Medtronic iPro 2, Medtronic); SMBG for 4 to 8 times/day	Fasting glucose ≤ 5.3 mmol/L, 1 h postprandial glucose ≤ 7.8 mmol/L
CONCEPTT trial 2017, Canada, England, Scotland, Spain, Italy, Ireland, USA	T1D	108/107	Real-time CGM (Medtronic iPro 2, Medtronic); SMBG for 4 times/day	Glucose 3.5 to 7.8 mmol/L
Wei 2016, China	GDM	51/55	Retrospective CGM (MiniMed Gold, Medtronic); SMBG for 4 times/day	Fasting glucose ≤ 90 mg/dL and 1 h postprandial glucose ≤ 140 mg/dL
Alfadhli 2016, Saudi Arabia	GDM	60/62	Real-time CGM (Guardian Real Time Continuous Glucose Monitoring System, Medtronic); SMBG for 4 times/day	Fasting glucose ≤ 95 mg/dL, 1 h postprandial glucose ≤ 140 mg/dL
Secher 2013, Denmark	T1D, T2D	79/75	Real-time CGM (Guardian Real Time Continuous Glucose Monitoring System, Medtronic); SMBG for 7 times/day	Fasting glucose ≤ 6 mmol/L, 1 h postprandial glucose ≤ 8 mmol/L
Murphy 2008, UK	T1D, T2D	38/33	Retrospective CGM (MiniMed Gold, Medtronic); SMBG for 4 times/day	Fasting glucose ≤ 5.5 mmol/L, 1 h postprandial glucose ≤ 7.8 mmol/L
Kestilä 2007, Finland	GDM	36/37	Retrospective CGM (MiniMed Gold, Medtronic); SMBG for 5 times/day	Fasting glucose ≤ 5.5 mmol/L, 1 h postprandial glucose ≤ 7.8 mmol/L

GDM, Gestational Diabetes Mellitus; CA, California; CGM, Continuous Glucose Monitoring; SMBG, Standard Self-Monitoring of Blood Glucose; USA, the United States of America; T1D, Type 1 Diabetes; T2D, Type 2 Diabetes.

In all included studies, the comparator arm was standard SMBG, with monitoring frequency ranging from 4 to 8 times/day (most commonly 4 times/day). Target glycemic thresholds were broadly comparable across studies but not identical. Most trials applied fasting targets between ≤90 and ≤95 mg/dL (or ≤5.0 to 5.3 mmol/L) and 1-hour postprandial targets of ≤140 mg/dL (or ≤7.8 to 8.0 mmol/L). A few studies used alternative target definitions, including 2-hour postprandial thresholds or a predefined glucose range (e.g., 70 to 140 mg/dL or 3.5 to 7.8 mmol/L), and one study did not report glycemic targets.

### Risk of bias assessment

**Figure 2:**
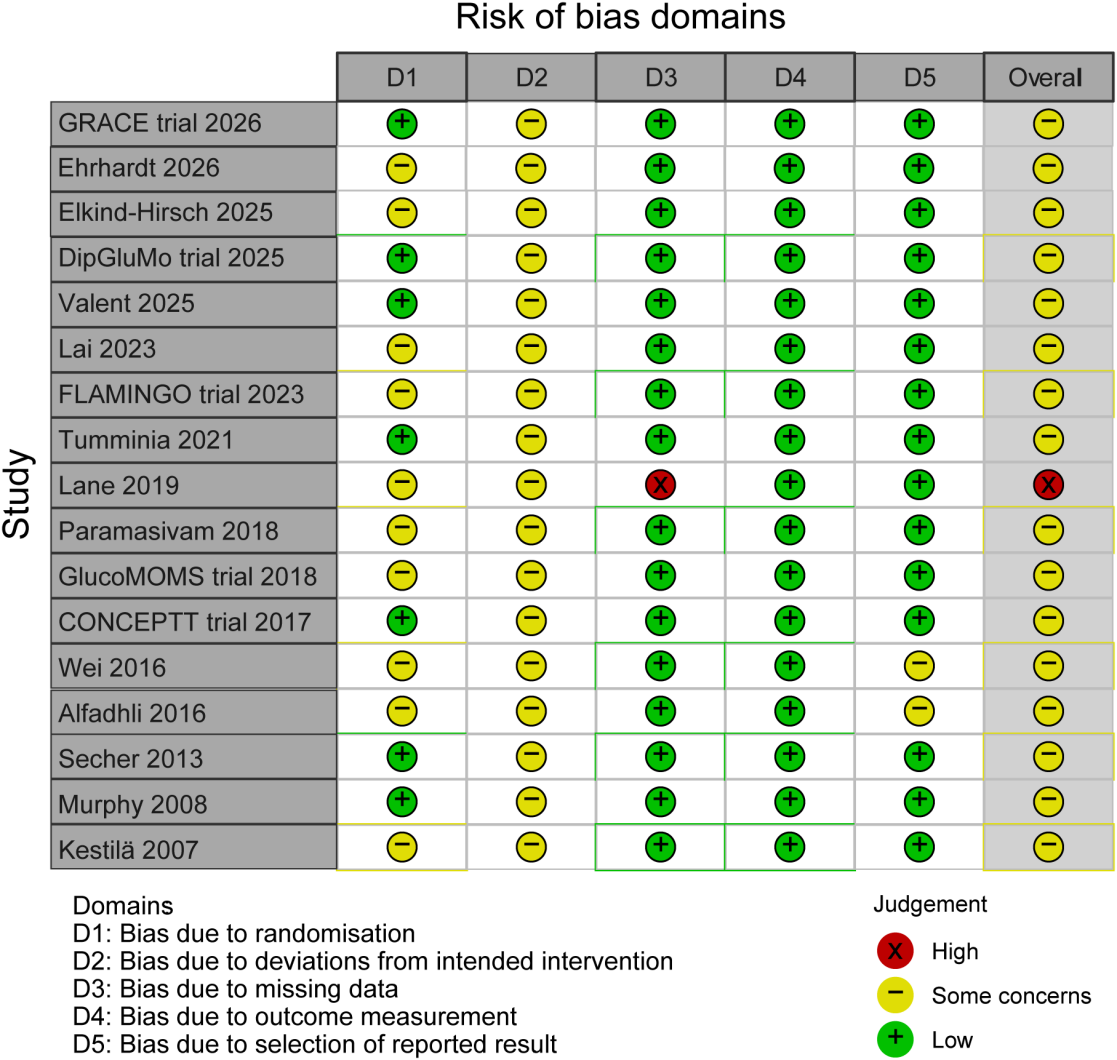
Risk of bias 2 of all included studies.

Sixteen trials were rated as having some concerns overall, reflecting the combination of open-label design and, in some cases, unclear randomization procedures. One trial [Lane et al. (29)] was rated as high risk overall due to substantial attrition and incomplete outcome data. These findings indicate that while the included RCTs generally demonstrated robust methodology in outcome measurement and handling of missing data, the open-label nature of CGM interventions and incomplete reporting of randomization procedures in some studies introduce some concerns that should be considered when interpreting the pooled effect estimates. Sensitivity analyses excluding the high-risk study did not materially alter the main findings. Detailed risk−of−bias assessments for individual studies are presented in **Figure 2**.

Potential publication bias was evaluated using Egger’s regression test for outcomes with a sufficient number of studies (**Supplementary Material 3**). No significant small-study effects were detected for LGA (Egger’s P = 0.4317), SGA (Egger’s P = 0.9410), neonatal hypoglycemia (Egger’s P = 0.8209), or NICU admission (Egger’s P = 0.6645). For neonatal hyperbilirubinemia, formal Egger’s testing was not performed because of the limited number of included studies. Therefore, a trim-and-fill analysis was conducted as an exploratory approach. After imputing three potentially missing studies, the adjusted pooled estimate was RR 0.80 (95% CI 0.60 to 1.09). The effect remained non-significant and directionally consistent with the primary analysis, suggesting that potential unpublished studies were unlikely to materially alter the conclusion for this outcome.

### Outcomes

#### LGA and SGA

**Figure 3:**
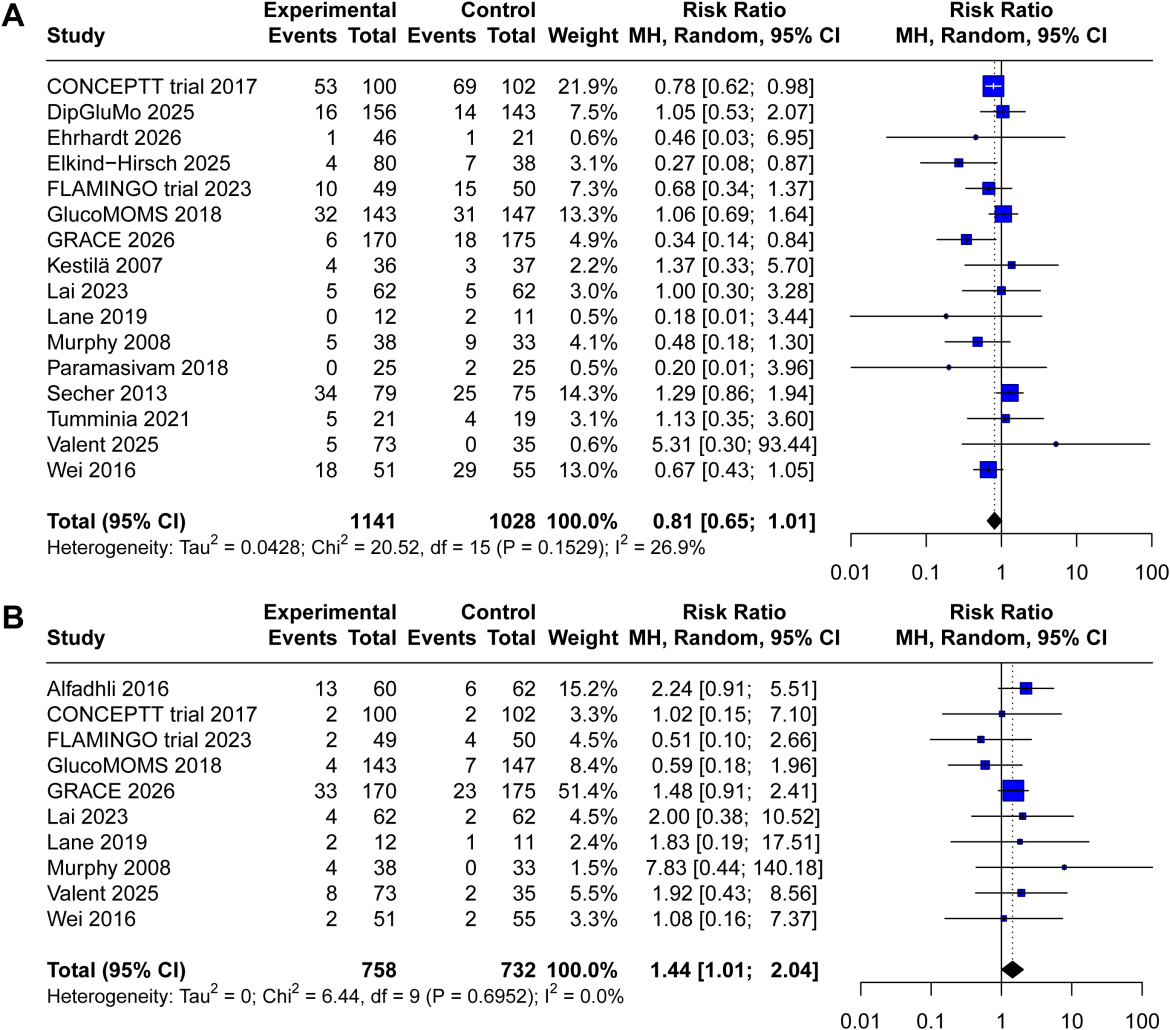
Forest plot comparing the effect of continuous glucose monitoring versus standard self−monitoring of blood glucose on **(A)** large for gestational age, **(B)** small for gestational age.

Sixteen trials reported data on LGA (1,141 in CGM group, 1028 in SMBG group). Pooled analysis demonstrated that CGM use was associated with a nonsignificant reduction in LGA compared with SMBG (RR 0.81, 95% CI 0.65 to 1.01, P = 0.06, I2 = 31%, **Figure 3A**). The forest plot shows that most individual studies favored CGM, however, the pooled estimate that did not reach statistical significance.

Data on SGA were available from ten included trials (758 in CGM group, 732 in SMBG group). Meta−analysis demonstrated that CGM use was associated with a significantly increased risk of SGA compared with SMBG (RR 1.44, 95% CI 1.01 to 2.04, P = 0.04, I2 = 0%, **Figure 3B**). The pooled estimate crossed the threshold for statistical significance.

#### Neonatal hypoglycemia, hyperbilirubinemia, and NICU admission

**Figure 4:**
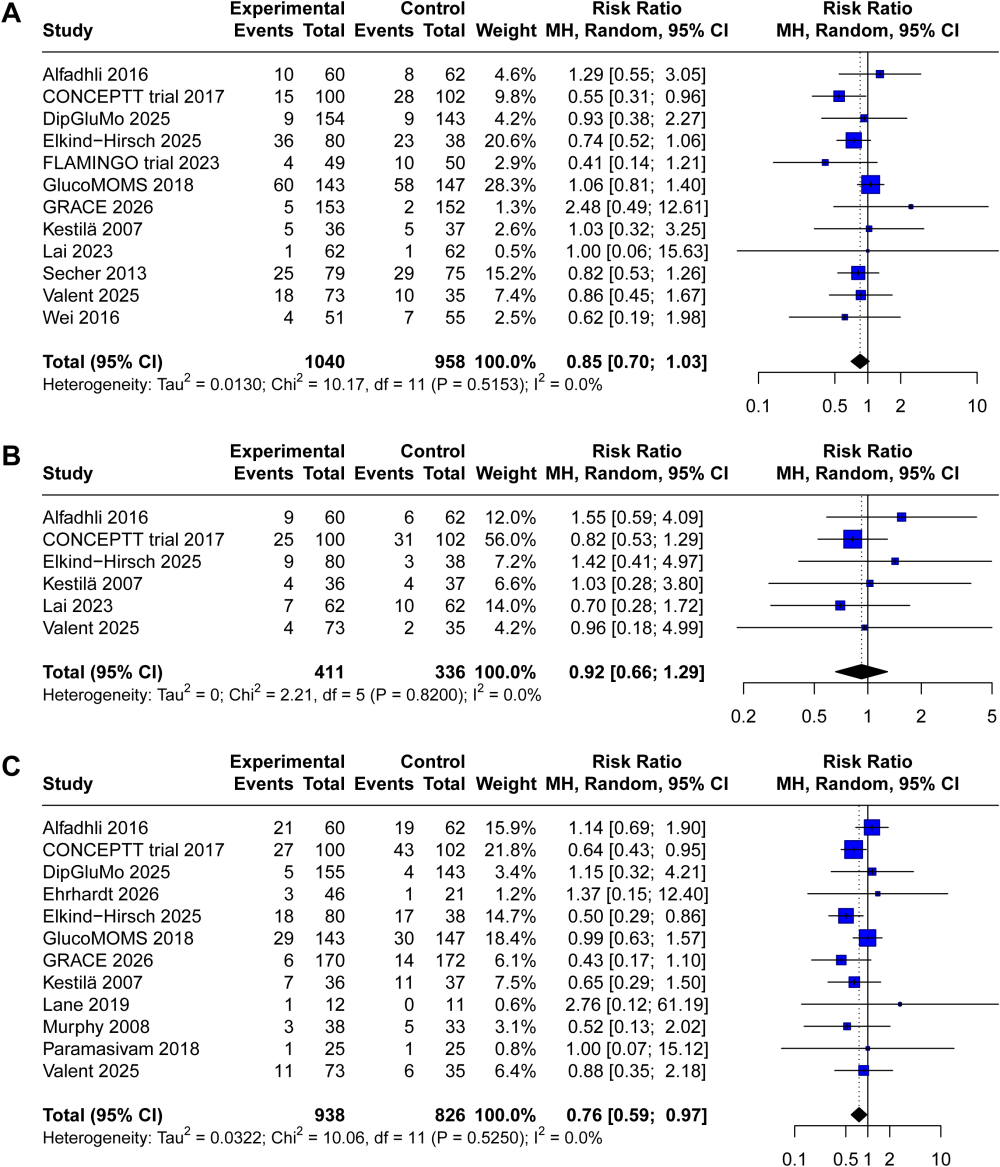
plot comparing the effect of continuous glucose monitoring versus standard self−monitoring of blood glucose on **(A)** neonatal hypoglycemia, **(B)** neonatal hyperbilirubinemia, **(C)** neonatal intensive care unit admission.

Neonatal hypoglycemia was reported in twelve included trials (1,040 in CGM group, 958 in SMBG group). Pooled results showed a trend toward reduced neonatal hypoglycemia in the CGM group compared with the SMBG group (RR 0.85, 95% CI 0.70 to 1.03, P = 0.10, I2 = 0%, **Figure 4A**). However, this difference did not reach statistical significance.

Six trials reported neonatal hyperbilirubinemia as an outcome (411 in CGM group, 336 in SMBG group). Meta−analysis revealed no significant difference between CGM and SMBG groups (RR 0.81, 95% CI 0.65 to 1.01, P = 0.64, I2 = 0%, **Figure 4B**). The forest plot shows substantial overlap of confidence intervals across studies, with point estimates distributed on both sides of unity, indicating no consistent effect of CGM on this outcome.

Admission to the NICU was reported from eleven included trials (938 in CGM group, 826 in SMBG group). Pooled analysis demonstrated that CGM use was associated with a significant reduction in NICU admission compared with SMBG (RR 0.75, 95% CI 0.59 to 0.97, P = 0.03, I2 = 0%, **Figure 4C**). The forest plot shows that the majority of studies favored CGM, with several individual trials demonstrating statistically significant reductions.

### Sensitivity and subgroup analyses

Sensitivity analyses were performed to evaluate the robustness of the pooled effects (**Supplementary Material 3**). For LGA, after sequential exclusion of single studies, pooled RRs ranged from 0.77 to 0.85. The association became statistically significant only when certain studies were omitted, indicating a consistent protective direction but limited statistical robustness. For SGA, pooled RRs ranged from 1.33 to 1.56 in leave-one-out analyses. Although the direction of effect remained unchanged, statistical significance was lost after exclusion of several individual studies, suggesting a fragile but directionally consistent signal. For neonatal hypoglycemia, leave-one-out estimates ranged from 0.78 to 0.90, and significance was generally not reached, except in isolated iterations. This indicates a stable direction of benefit but non-robust significance. For neonatal hyperbilirubinemia, sequential exclusion of individual studies produced similar results (RR range 0.86 to 1.07), with all confidence intervals crossing unity, supporting a robust null finding. For NICU admission, pooled RRs ranged from 0.70 to 0.82 in leave-one-out analyses. The protective direction persisted throughout, but statistical significance was attenuated after removal of specific influential studies, indicating moderate dependence on individual trials. Overall, sensitivity analyses showed that the direction of effects was generally stable across outcomes. However, the statistical significance of LGA, SGA, hypoglycemia, and NICU admission was variably sensitive to omission of individual studies, whereas the null result for hyperbilirubinemia was consistently robust.

Subgroup analyses were performed by types of CGM and diabetes. For LGA, effect estimates were comparable between CGM modality subgroups (RT-CGM: RR 0.77, 95% CI 0.51 to 1.17; other methods: RR 0.80, 95% CI 0.60 to 1.06). By diabetes type, a significant reduction was observed in GDM (RR 0.67, 95% CI 0.51 to 0.89), whereas no significant effect was found in T1D/T2D (RR 0.94, 95% CI 0.71 to 1.24). For SGA, RT-CGM was associated with a higher risk (RR 1.64, 95% CI 1.11 to 2.41), while other CGM methods were not (RR 0.79, 95% CI 0.34 to 1.81). A similar pattern was observed by diabetes type: the risk was increased in GDM (RR 1.54, 95% CI 1.06 to 2.25), but not in T1D/T2D (RR 0.94, 95% CI 0.33 to 2.68). For neonatal hypoglycemia, RT-CGM showed a significant protective association (RR 0.79, 95% CI 0.64 to 0.98), whereas other methods did not (RR 0.87, 95% CI 0.57 to 1.35). Stratified by diabetes type, neither subgroup reached statistical significance (GDM: RR 0.82, 95% CI 0.63 to 1.05; T1D/T2D: RR 0.83, 95% CI 0.58 to 1.19). For hyperbilirubinemia, subgroup estimates were generally consistent across CGM modality and diabetes type, without meaningful between-subgroup differences. For NICU admission, both CGM modality subgroups showed a non-significant trend toward reduced risk (RT-CGM: RR 0.73, 95% CI 0.53 to 1.01; other methods: RR 0.87, 95% CI 0.59 to1.26). Similar non-significant reductions were observed by diabetes type (GDM: RR 0.76, 95% CI 0.53 to 1.09; T1D/T2D: RR 0.76, 95% CI 0.52 to 1.11).

Overall, subgroup findings suggest potential effect modification by diabetes type for fetal growth outcomes (LGA and SGA), with more pronounced associations in GDM, while results for hypoglycemia and NICU admission were directionally favorable but largely not statistically significant across strata.

## Discussion

### Principal findings

In this systematic review and meta−analysis of seventeen RCTs including 2,349 women with GDM, CGM use during pregnancy complicated by diabetes was associated with differential effects across neonatal outcomes, and subgroup analyses suggested clinically relevant heterogeneity by monitoring modality and diabetes type. For fetal growth, CGM was associated with a significant reduction in LGA in women with GDM, while no clear effect was observed in women with T1D/T2D. In contrast, the risk of SGA appeared increased in RT-CGM and GDM subgroups, suggesting a possible shift in fetal growth distribution when tighter glycemic control is achieved in selected populations. For neonatal hypoglycemia, RT-CGM showed a significant protective association, although this was not consistently significant across diabetes-type strata. For NICU admission, effect estimates were directionally favorable but not statistically significant across subgroups. Hyperbilirubinemia results were broadly similar across strata, with no clear subgroup-specific pattern.

Taken together, these findings indicate that CGM-related benefits may be outcome-specific and context-dependent, with stronger signals in GDM for growth-related endpoints and in RT-CGM for hypoglycemia reduction.

### Comparison with existing literature

The present meta-analysis provides a comprehensive evaluation of real-time continuous glucose monitoring effects on neonatal outcomes across the spectrum of diabetes in pregnancy, incorporating recent randomized controlled trials and offering several novel insights not previously reported. Our findings demonstrate that CGM use is associated with a significant reduction in LGA infants, neonatal hypoglycemia, and NICU admissions, while concurrently identifying an increased risk of SGA, a signal that has been inconsistently captured in prior meta-analyses.

Our findings that CGM reduces the risk of LGA infants and neonatal hypoglycemia are largely consistent with high-quality evidence from landmark trials. The CONCEPTT trial demonstrated that CGM use throughout pregnancy in women with type 1 diabetes significantly lowered the incidence of LGA and neonatal hypoglycemia requiring intravenous dextrose (27). Similarly, the recent GRACE trial and Elkind-Hirsch et al. reported significant reductions in LGA rates among users of CGM systems in GDM populations (10, 34). Burk et al. (8), in the largest and most contemporary meta-analysis to date, similarly reported that continuous CGM use in T1D significantly reduced LGA, with high-certainty evidence. However, our analysis extends these findings by demonstrating that this benefit is contingent upon continuous rather than intermittent CGM use, a distinction not explicitly addressed in prior syntheses. When RCTs employing intermittent CGM in T1D were pooled separately, no LGA reduction was observed, underscoring the importance of uninterrupted glucose monitoring throughout gestation. In contrast, the systematic review by Chang et al. (13), which included mixed diabetes types (T1D, T2D, GDM), reported a small but significant reduction in neonatal birth weight but no significant effect on macrosomia. Our subgroup analysis by diabetes type and CGM duration clarifies this heterogeneity, revealing that the LGA benefit is primarily driven by GDM pregnancies with continuous RT-CGM. Notably, the DipGluMo trial reported no difference in LGA or macrosomia between CGM and SMBG in a low-risk GDM cohort, suggesting that any potential benefit in GDM may be confined to higher-risk populations or require different TIR targets than those currently recommended (33).

Our finding that RT-CGM reduces neonatal hypoglycemia and NICU admissions in pregnant women with diabetes corroborates the CONCEPTT trial and the subsequent Cochrane review by Jones et al. (36), which reported reduced neonatal hypoglycemia with CGM in pre-existing diabetes. However, both the Cochrane review and the broader meta-analysis by Chang et al. failed to detect significant effects on these outcomes when T1D, T2D, and GDM were analyzed collectively, likely due to dilution of effect by the inclusion of lower-risk populations and heterogeneous CGM regimens (13, 36). Moreover, this discrepancy may also stem from our inclusion of more recent, adequately powered RCTs that employed continuous, real-time feedback rather than earlier studies that used intermittent or retrospective CGM.

A novel and concerning finding in our meta-analysis is the increased risk of SGA associated with RT-CGM use. This association has not been systematically reported in prior meta-analyses. Chang et al. (2022) did not report SGA as an outcome, while Garcia-Moreno et al. (14) found no difference in SGA across six GDM studies, though confidence intervals were wide and did not exclude harm. The TOBOGM trial of early GDM treatment reported increased SGA with tighter glycemic control, raising the possibility that CGM-facilitated intensification may inadvertently increase SGA risk, particularly when treatment targets are extrapolated from T1D populations without adjustment for the lower baseline glycemia in GDM and T2D (37).

Our meta-analysis advances the existing literature in several respects. First, unlike the broad “diabetes in pregnancy” approach taken by Chang et al. (13) and Rizos et al. (9), we performed distinct subgroup analyses by diabetes type and CGM duration, revealing effect modification masked in pooled analyses. Second, we incorporated the most recent RCTs in GDM (10, 35), which were not included in prior syntheses and collectively temper earlier optimism regarding CGM efficacy in low-risk GDM.

### Clinical implications

These results support a more tailored implementation of CGM in diabetic pregnancy rather than a one-size-fits-all approach. First, the apparent LGA benefit in GDM suggests that CGM may be particularly useful in pregnancies where prevention of fetal overgrowth is a primary therapeutic goal. Second, the potential increase in SGA in specific subgroups underscores the need for balanced glycemic targets, careful nutritional counseling, and close fetal growth surveillance when intensifying glucose management. Third, the favorable signal for neonatal hypoglycemia with RT-CGM suggests that real-time data and immediate feedback may offer additional clinical value beyond intermittent or less interactive CGM approaches.

From a practice standpoint, CGM interpretation should be integrated with obstetric risk assessment, fetal biometry, and individualized glucose targets. Future care pathways may benefit from stratifying patients by diabetes phenotype and baseline risk, with dynamic adjustment of glycemic goals during gestation to optimize both maternal control and fetal growth.

### Limitations

Several limitations should be considered when interpreting our results. First, although only RCTs were included, substantial heterogeneity exists across included studies regarding CGM type, duration of use, timing of initiation, and glycemic targets. This clinical and methodological diversity necessitated random-effects models and limits the precision of pooled estimates. Second, blinding of participants and clinicians was not feasible, which may have influenced clinical decision−making, although neonatal outcomes are largely objective. Third, definitions of neonatal outcomes, particularly hypoglycemia, varied among studies, potentially introducing measurement variability. Finally, long−term neurodevelopmental and metabolic outcomes of infants were not assessed and remain an important area for future research. Finally, the majority of participants across trials were of white European origin, highlighting a need for more diverse data to assess the impact of CGM across different ethnicities.

## Conclusion

In conclusion, this meta-analysis suggests that the impact of continuous glucose monitoring in pregnancies complicated by diabetes is outcome- and population-dependent. Subgroup findings indicate a significant reduction in LGA among women with GDM and a potential reduction in neonatal hypoglycemia with RT-CGM, while effects on NICU admission were directionally favorable but not statistically significant. Notably, the observed increase in SGA in RT-CGM and GDM subgroups highlights the need to balance tighter glycemic control against potential risks of restricted fetal growth. Overall, these results support individualized CGM-based management strategies tailored to diabetes type and fetal growth patterns. Further well-designed, adequately powered studies are needed to clarify subgroup-specific effects and optimize glycemic targets that maximize neonatal benefit while minimizing harm.

## Data Availability

The original contributions presented in the study are included in the article/**Supplementary Material**. Further inquiries can be directed to the corresponding author.

## References

[1] Sweeting A Hare MJ de Jersey SJ Shub AL Zinga J Foged C . Australasian Diabetes in Pregnancy Society (ADIPS) 2025 consensus recommendations for the screening, diagnosis and classification of gestational diabetes. Med J Aust. (2025) 223:161–7. doi: 10.5694/mja2.52696, PMID: 40544364 PMC12318493

[2] American Diabetes Association Professional Practice Committee . Management of Diabetes in Pregnancy: Standards of Care in Diabetes-2025. Diabetes Care. (2025) 48:S306–S320. doi: 10.2337/dc25-S015, PMID: 39651985 PMC11635054

[3] McIntyre HD Catalano P Zhang C Desoye G Mathiesen ER Damm P . Gestational diabetes mellitus. Nat Rev Dis Primers. (2019) 5:47. doi: 10.1038/s41572-019-0098-8, PMID: 31296866

[4] Ye W Luo C Huang J Li C Liu Z Liu F . Gestational diabetes mellitus and adverse pregnancy outcomes: systematic review and meta-analysis. Bmj. (2022) 377:e067946. doi: 10.1136/bmj-2021-067946, PMID: 35613728 PMC9131781

[5] Hashimoto K Koga M . Indicators of glycemic control in patients with gestational diabetes mellitus and pregnant women with diabetes mellitus. World J Diabetes. (2015) 6:1045–56. doi: 10.4239/wjd.v6.i8.1045, PMID: 26240701 PMC4515444

[6] Kintiraki E Goulis DG . Gestational diabetes mellitus: Multi-disciplinary treatment approaches. Metabolism. (2018) 86:91–101. doi: 10.1016/j.metabol.2018.03.025, PMID: 29627447

[7] American Diabetes Association Professional Practice Committee . Diabetes Technology: Standards of Care in Diabetes-2025. Diabetes Care. (2025) 48:S146–S166. doi: 10.2337/dc25-S007, PMID: 39651978 PMC11635043

[8] Burk J Ross GP Hernandez TL Colagiuri S Sweeting A . Evidence for improved glucose metrics and perinatal outcomes with continuous glucose monitoring compared to self-monitoring in diabetes during pregnancy. Am J Obstet Gynecol. (2025) 233:162–75. doi: 10.1016/j.ajog.2025.04.010, PMID: 40216177

[9] Rizos EC Markozannes G Charitakis N Filis P Stoimeni AE Nørgaard K . Continuous glucose monitoring in type 1 diabetes, type 2 diabetes, and diabetes during pregnancy: A systematic review with meta-analysis of randomized controlled trials. Diabetes Technol Ther. (2025) 27:537–52. doi: 10.1089/dia.2024.0599, PMID: 40049630

[10] Linder T Dressler-Steinbach I Wegener S Schellong K Schmidt S Eppel D . Winzeler B et al: Glycaemic control and pregnancy outcomes with real-time continuous glucose monitoring in gestational diabetes (GRACE): an open-label, multicentre, multinational, randomised controlled trial. Lancet Diabetes Endocrinol. (2026) 14:50–61. doi: 10.1016/S2213-8587(25)00288-8, PMID: 41308662

[11] Valent AM Rickert M Pagan CH Ward L Dunn E Rincon M . Real-time continuous glucose monitoring in pregnancies with gestational diabetes mellitus: A randomized controlled trial. Diabetes Care. (2025) 48:1581–8. doi: 10.2337/dc25-0115, PMID: 40730104 PMC12368369

[12] Voormolen DN DeVries JH Sanson RME Heringa MP de Valk HW Kok M . Brouwer TCB et al: Continuous glucose monitoring during diabetic pregnancy (GlucoMOMS): A multicentre randomized controlled trial. Diabetes Obes Metab. (2018) 20:1894–902. doi: 10.1111/dom.13310, PMID: 29603547

[13] Chang VYX Tan YL Ang WHD Lau Y . Effects of continuous glucose monitoring on maternal and neonatal outcomes in perinatal women with diabetes: A systematic review and meta-analysis of randomized controlled trials. Diabetes Res Clin Pract. (2022) 184:109192. doi: 10.1016/j.diabres.2022.109192, PMID: 35032563

[14] García-Moreno RM Benítez-Valderrama P Barquiel B González Pérez-de-Villar N Hillman N Lora Pablos D . Efficacy of continuous glucose monitoring on maternal and neonatal outcomes in gestational diabetes mellitus: a systematic review and meta-analysis of randomized clinical trials. Diabetes Med. (2022) 39:e14703. doi: 10.1111/dme.14703, PMID: 34564868

[15] Alfadhli E Osman E Basri T . Use of a real time continuous glucose monitoring system as an educational tool for patients with gestational diabetes. Diabetol Metab Syndr. (2016) 8:48. doi: 10.1186/s13098-016-0161-5, PMID: 27468313 PMC4962392

[16] Page MJ McKenzie JE Bossuyt PM Boutron I Hoffmann TC Mulrow CD . The PRISMA 2020 statement: an updated guideline for reporting systematic reviews. BMJ. (2021) 372:n71. doi: 10.1186/s13643-021-01626-4, PMID: 33782057 PMC8005924

[17] Sterne JAC Savović J Page MJ Elbers RG Blencowe NS Boutron I . RoB 2: a revised tool for assessing risk of bias in randomised trials. Bmj. (2019) 366:l4898. doi: 10.1136/bmj.l4898, PMID: 31462531

[18] Higgins JP Thompson SG Deeks JJ Altman DG . Measuring inconsistency in meta-analyses. Bmj. (2003) 327:557–60. doi: 10.1136/bmj.327.7414.557, PMID: 12958120 PMC192859

[19] Egger M Davey Smith G Schneider M Minder C . Bias in meta-analysis detected by a simple, graphical test. Bmj. (1997) 315:629–34. doi: 10.1136/bmj.315.7109.629, PMID: 9310563 PMC2127453

[20] Duval S Tweedie R . Trim and fill: A simple funnel-plot-based method of testing and adjusting for publication bias in meta-analysis. Biometrics. (2000) 56:455–63. doi: 10.1111/j.0006-341X.2000.00455.x, PMID: 10877304

[21] Balduzzi S Rücker G Schwarzer G . How to perform a meta-analysis with R: a practical tutorial. Evid Based Ment Health. (2019) 22:153–60. doi: 10.1136/ebmental-2019-300117, PMID: 31563865 PMC10231495

[22] McGuinness LA Higgins JPT . Risk-of-bias VISualization (robvis): An R package and Shiny web app for visualizing risk-of-bias assessments. Res Synth Methods. (2021) 12:55–61. doi: 10.1002/jrsm.1411, PMID: 32336025

[23] Kestilä KK Ekblad UU Rönnemaa T . Continuous glucose monitoring versus self-monitoring of blood glucose in the treatment of gestational diabetes mellitus. Diabetes Res Clin Pract. (2007) 77:174–9. doi: 10.1016/j.diabres.2006.12.012, PMID: 17234297

[24] Murphy HR Rayman G Lewis K Kelly S Johal B Duffield K . Effectiveness of continuous glucose monitoring in pregnant women with diabetes: randomised clinical trial. Bmj. (2008) 337:a1680–0. doi: 10.1136/bmj.a1680, PMID: 18818254 PMC2563261

[25] Secher AL Ringholm L Andersen HU Damm P Mathiesen ER . The effect of real-time continuous glucose monitoring in pregnant women with diabetes. Diabetes Care. (2013) 36:1877–83. doi: 10.2337/dc12-2360, PMID: 23349548 PMC3687305

[26] Wei Q Sun Z Yang Y Yu H Ding H Wang S . Effect of a CGMS and SMBG on maternal and neonatal outcomes in gestational diabetes mellitus: a randomized controlled trial. Sci Rep. (2016) 6:19920. doi: 10.1038/srep19920, PMID: 26814139 PMC4728693

[27] Feig DS Donovan LE Corcoy R Murphy KE Amiel SA Hunt KF . Continuous glucose monitoring in pregnant women with type 1 diabetes (CONCEPTT): a multicentre international randomised controlled trial. Lancet. (2017) 390:2347–59. doi: 10.1016/S0140-6736(17)32400-5, PMID: 28923465 PMC5713979

[28] Paramasivam SS Chinna K Singh AKK Ratnasingam J Ibrahim L Lim LL . Continuous glucose monitoring results in lower HbA1c in Malaysian women with insulin-treated gestational diabetes: a randomized controlled trial. Diabetic Med. (2018) 35:1118–29. doi: 10.1111/dme.13649, PMID: 29663517

[29] Lane AS Mlynarczyk MA de Veciana M Green LM Baraki DI Abuhamad AZ . Real-time continuous glucose monitoring in gestational diabetes: A randomized controlled trial. Am J Perinatology. (2019) 36:891–7. doi: 10.1055/s-0039-1678733, PMID: 30818406

[30] Tumminia A Milluzzo A Festa C Fresa R Pintaudi B Scavini M . Efficacy of flash glucose monitoring in pregnant women with poorly controlled pregestational diabetes (FlashMom): A randomized pilot study. Nutrition Metab Cardiovasc Dis. (2021) 31:1851–9. doi: 10.1016/j.numecd.2021.03.013, PMID: 33975741

[31] Lai M Weng J Yang J Gong Y Fang F Li N . Effect of continuous glucose monitoring compared with self-monitoring of blood glucose in gestational diabetes patients with HbA1c<6%: a randomized controlled trial. Front Endocrinol. (2023) 14:1174239. doi: 10.3389/fendo.2023.1174239, PMID: 37152928 PMC10155499

[32] Majewska A Stanirowski PJ Tatur J Wojda B Radosz I Wielgos M . Flash glucose monitoring in gestational diabetes mellitus (FLAMINGO): a randomised controlled trial. Acta Diabetologica. (2023) 60:1171–7. doi: 10.1007/s00592-023-02091-2, PMID: 37160787 PMC10359198

[33] Amylidi-Mohr S Zennaro G Schneider S Raio L Mosimann B Surbek D . Continuous glucose monitoring in the management of gestational diabetes in Switzerland (DipGluMo): an open-label, single-centre, randomised, controlled trial. Lancet Diabetes Endocrinol. (2025) 13:591–9. doi: 10.1016/S2213-8587(25)00063-4, PMID: 40441173

[34] Elkind-Hirsch K Armatta M Griffen C Welsh JB Veillon E Guedry S . Continuous glucose monitoring in early gestational diabetes improves maternal and neonatal outcomes—The Steady Sugar trial. Diabetes Obes Metab. (2025) 28:691–700. doi: 10.1111/dom.70254, PMID: 41178671 PMC12673445

[35] Ehrhardt N Fonda SJ Mandava P Abdalla G Fay E . A randomized controlled trial of real-time continuous glucose monitoring (RT-CGM) for self-management of gestational diabetes. Am J Obstet Gynecol MFM. (2026) 8:101878. doi: 10.1016/j.ajogmf.2025.101878, PMID: 41419058

[36] Jones LV Ray A Moy FM Buckley BS . Techniques of monitoring blood glucose during pregnancy for women with pre-existing diabetes. Cochrane Database Syst Rev. (2019) 5:Cd009613. doi: 10.1002/14651858.CD009613.pub4, PMID: 31120549 PMC6532756

[37] Simmons D Nema J Parton C Vizza L Robertson A Rajagopal R . The treatment of booking gestational diabetes mellitus (TOBOGM) pilot randomised controlled trial. BMC Pregnancy Childbirth. (2018) 18:151. doi: 10.1186/s12884-018-1809-y, PMID: 29747594 PMC5946423

