# Supplementary material for: The effect of continuous glucose monitoring on neonatal outcomes in pregnant women with diabetes
Source: Front Endocrinol (Lausanne). 2026 Mar 25;17:1815133. doi: 10.3389/fendo.2026.1815133 (PMC13056827; doi:10.3389/fendo.2026.1815133)
Supplement: Supplementary file 2 [file DataSheet2.docx]

**Supplementary Material 2: Searching strategies in Pubmed**

**Pubmed 118**

#1 Pregnancy [MeSH Terms] OR pregnancy [Title/Abstract] OR pregnant [Title/Abstract] OR Pregnancies [Title/Abstract]

#2 Continuous Glucose Monitoring [MeSH Terms] OR Continuous Glucose Monitoring [Title/Abstract]

#3 Diabetes Mellitus [MeSH Terms] OR Diabetes, Gestational [MeSH Terms] OR Diabetes [Title/Abstract]

#4 randomized controlled trial [MeSH Terms] OR randomized [Title/Abstract] OR randomised [Title/Abstract]

#1 AND #2 AND #3 AND #4

**Embase 93**

#1 ‘Pregnancy’:ti,ab,kw OR ‘pregnant’:ti,ab,kw OR ' pregnancy '/exp OR ‘Pregnancies’:ti,ab,kw

#2 ' Continuous Glucose Monitoring ':ti,ab,kw OR ‘CGM’:ti,ab,kw

#3 ' Diabetes Mellitus ':ti,ab,kw OR ' Diabetes '/exp

#4 'randomized controlled trial'/de OR 'randomized controlled trial'/exp

#1 AND #2 AND #3 AND #4

**Scopus 149**

#1 TITLE-ABS-KEY (Pregnancy) OR TITLE-ABS-KEY (pregnant) OR TITLE-ABS-KEY (Pregnancies)

#2 TITLE-ABS-KEY (Continuous Glucose Monitoring) OR TITLE-ABS-KEY (CGM)

#3 TITLE-ABS-KEY (Diabetes)

#4 TITLE-ABS-KEY (randomized) OR TITLE-ABS-KEY (random) OR TITLE-ABS-KEY (randomised)

#1 AND #2 AND #3 AND #4

**Cochrane Library 276**

#1 (Pregnancy):ti,ab,kw OR (Pregnancies):ti,ab,kw OR (pregnant):ti,ab,kw

#2 (Continuous Glucose Monitoring):ti,ab,kw OR (CGM):ti,ab,kw

#3 (Diabetes):ti,ab,kw

#4 (randomized):ti,ab,kw OR (randomised):ti,ab,kw OR (random):ti,ab,kw

#1 AND #2 AND #3 AND #4
