# Supplementary material for: The effect of continuous glucose monitoring on neonatal outcomes in pregnant women with diabetes
Source: Front Endocrinol (Lausanne). 2026 Mar 25;17:1815133. doi: 10.3389/fendo.2026.1815133 (PMC13056827; doi:10.3389/fendo.2026.1815133)
Supplement: Supplementary file 3 [file DataSheet3.docx]

**Supplementary Material 3:** Publication bias assessment by funnel plot and Egger’s test, forest plot after trim and fill method, forest plot for sensitivity and subgroup analysis


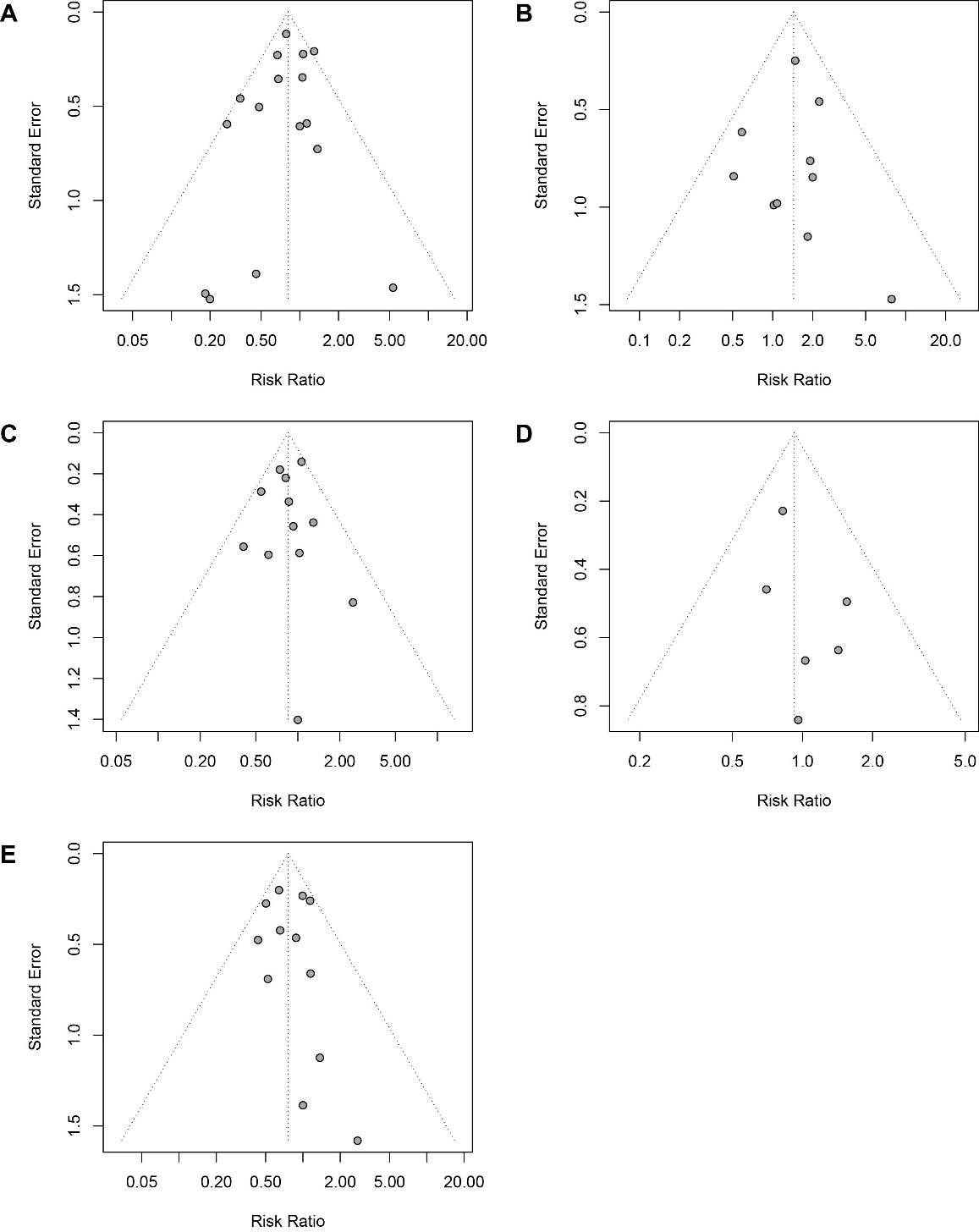


Figure 1: Funnel plot for (A) large for gestational age, Egger’s test P=0.4317; (B) small for gestational age, Egger’s test P=0.9410; (C) neonatal hypoglycemia, Egger’s test P=0.8209; (D) neonatal hyperbilirubinemia; (E) admission to the neonatal intensive care unit, Egger’s test P=0.6645


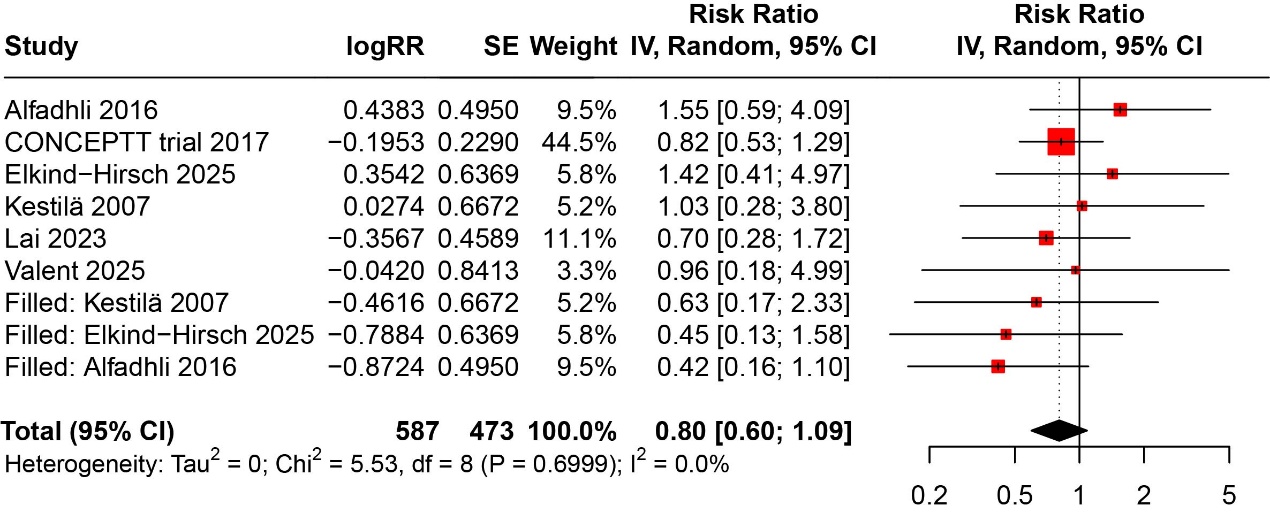


Figure 2: Forest plot for neonatal hyperbilirubinemia after trim and fill method


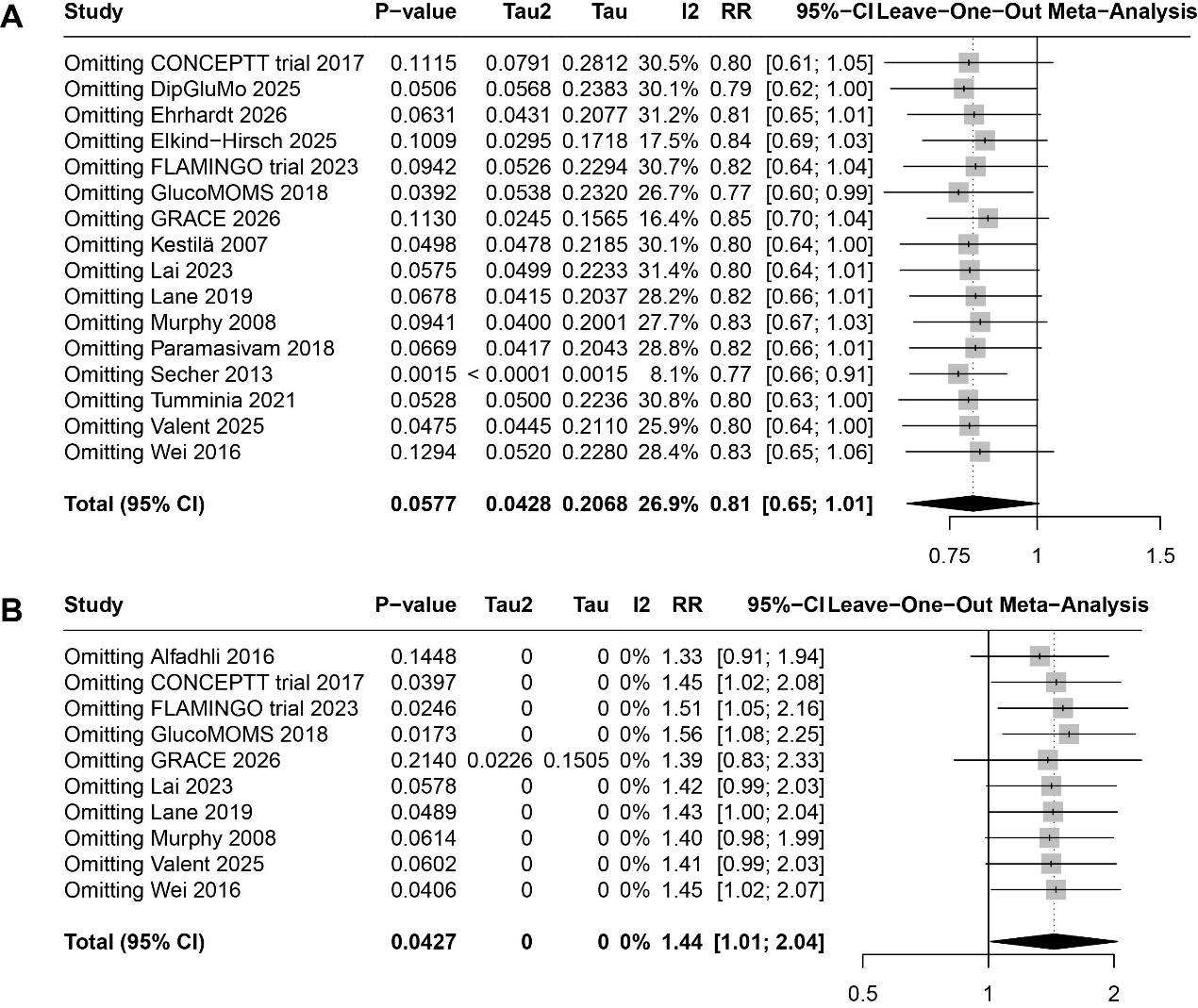


Figure 3: Forest plot for sensitivity analysis, (A) large for gestational age; (B) small for gestational age, Egger’s test P=0.9410


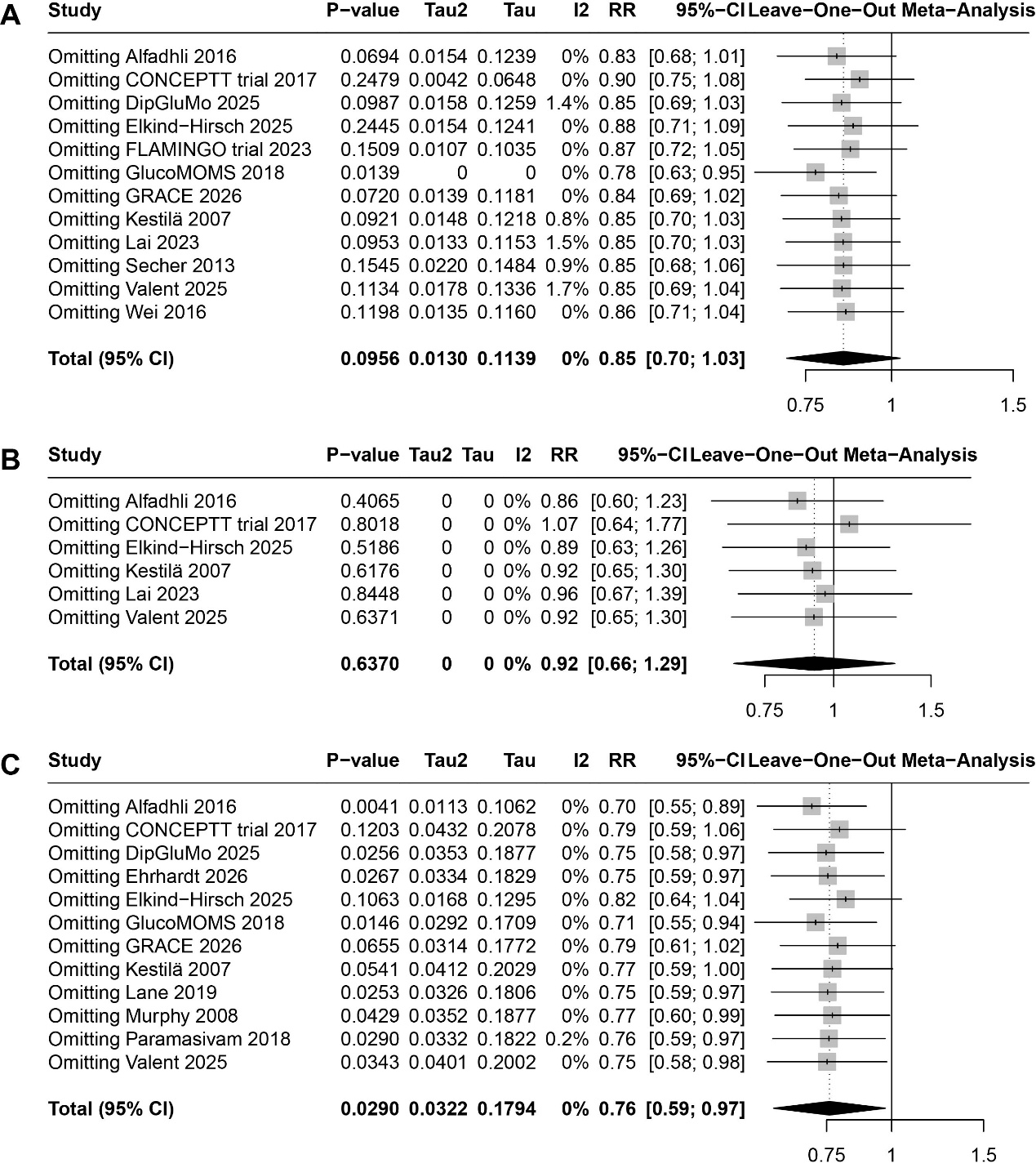


Figure 4: Forest plot for sensitivity analysis, (A) neonatal hypoglycemia; (B) neonatal hyperbilirubinemia; (C) admission to the neonatal intensive care unit


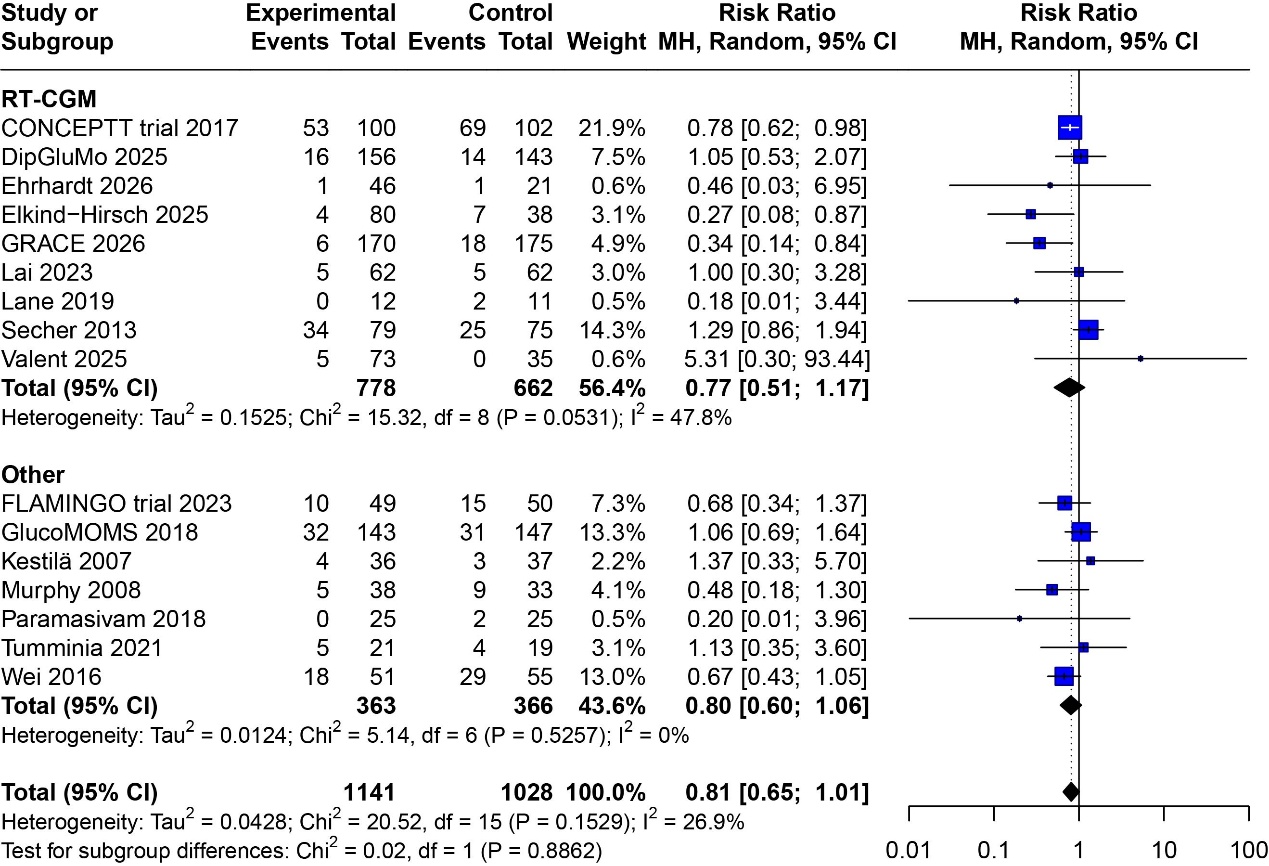


Figure 5: Forest plot for subgroup analysis for large for gestational age, real-time CGM versus other CGM


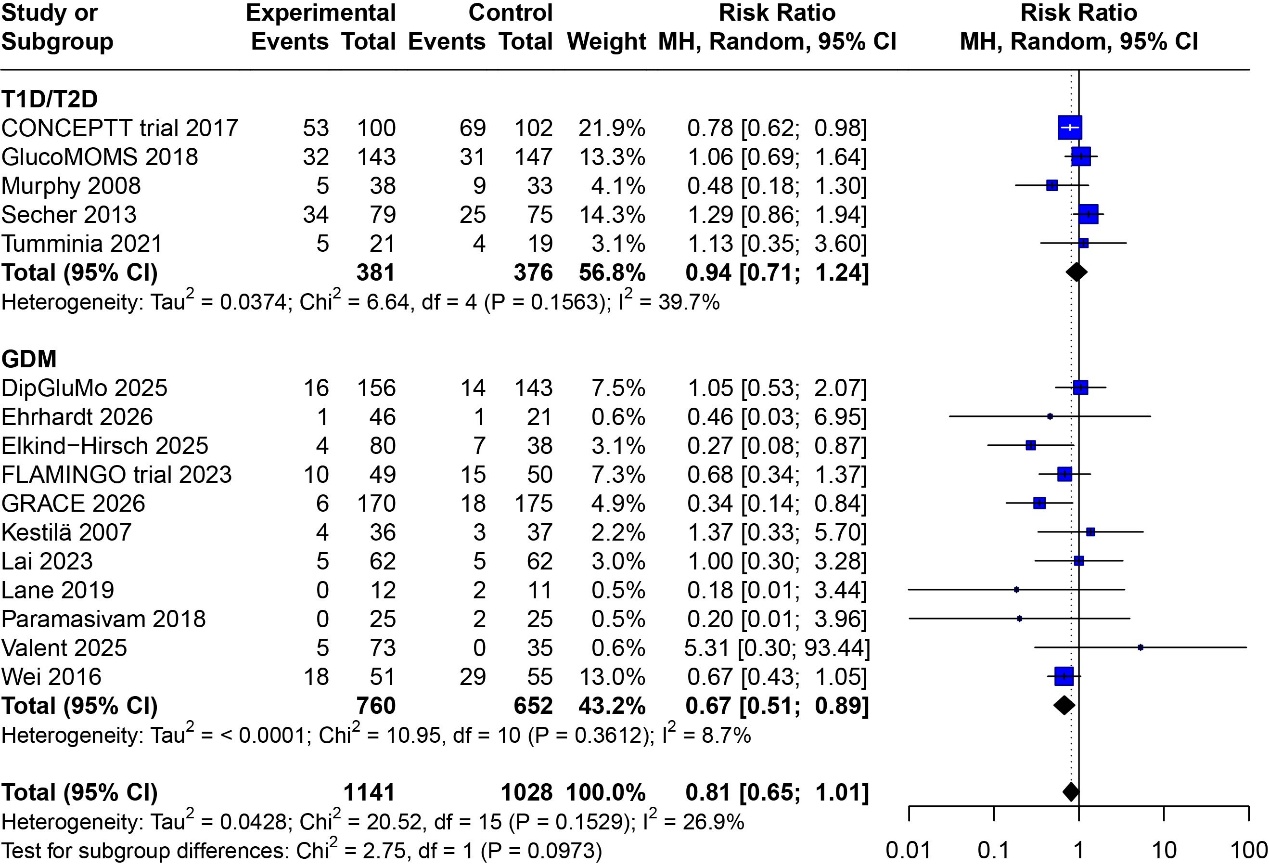


Figure 6: Forest plot for subgroup analysis for large for gestational age, T1D/T2D versus GDM


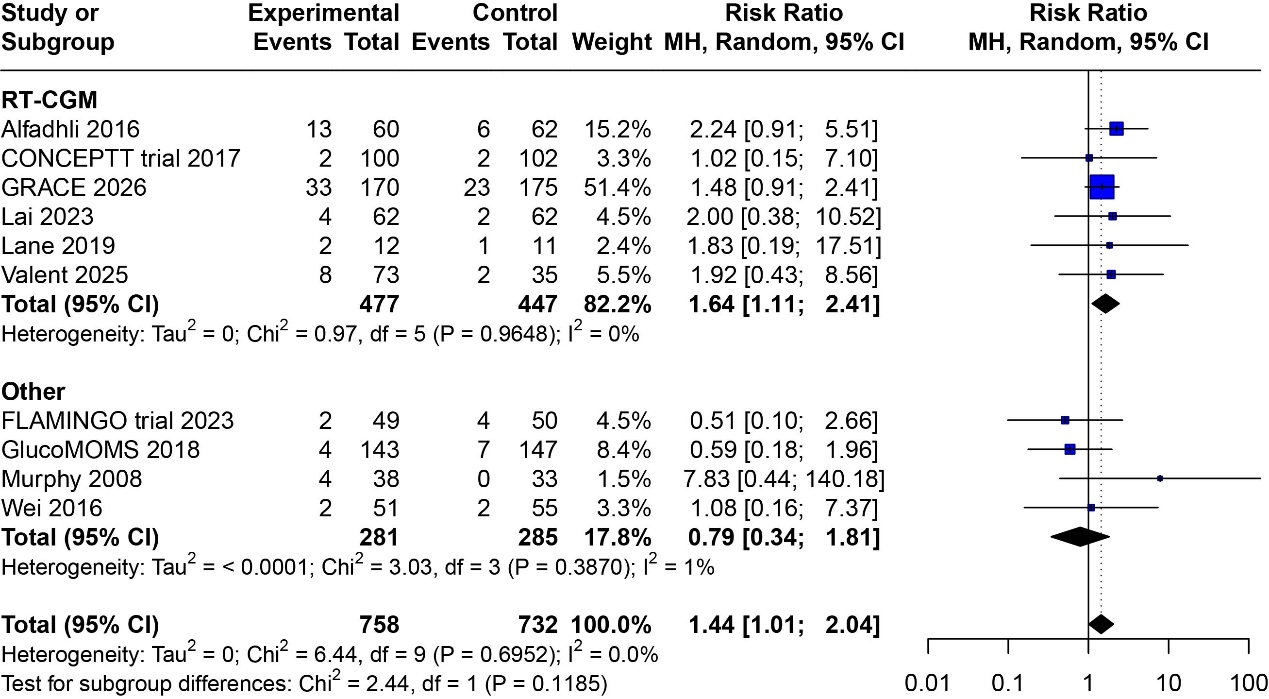


Figure 7: Forest plot for subgroup analysis for small for gestational age, real-time CGM versus other CGM


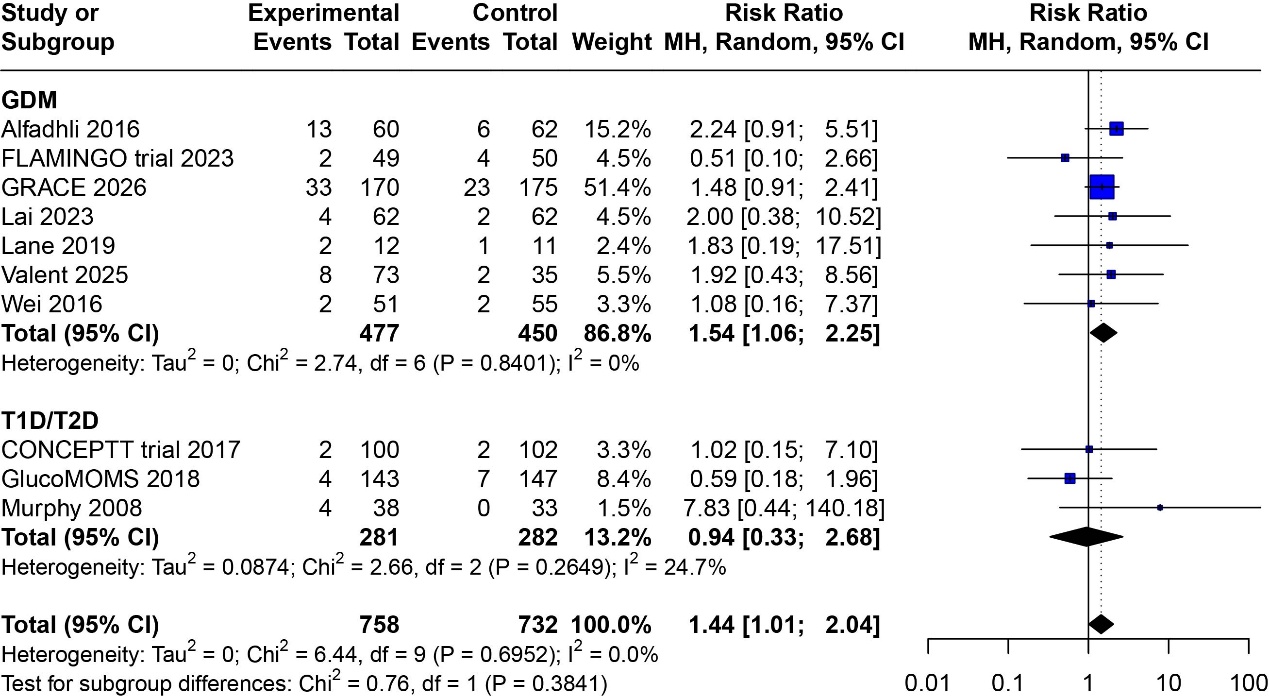


Figure 8: Forest plot for subgroup analysis for small for gestational age, T1D/T2D versus GDM


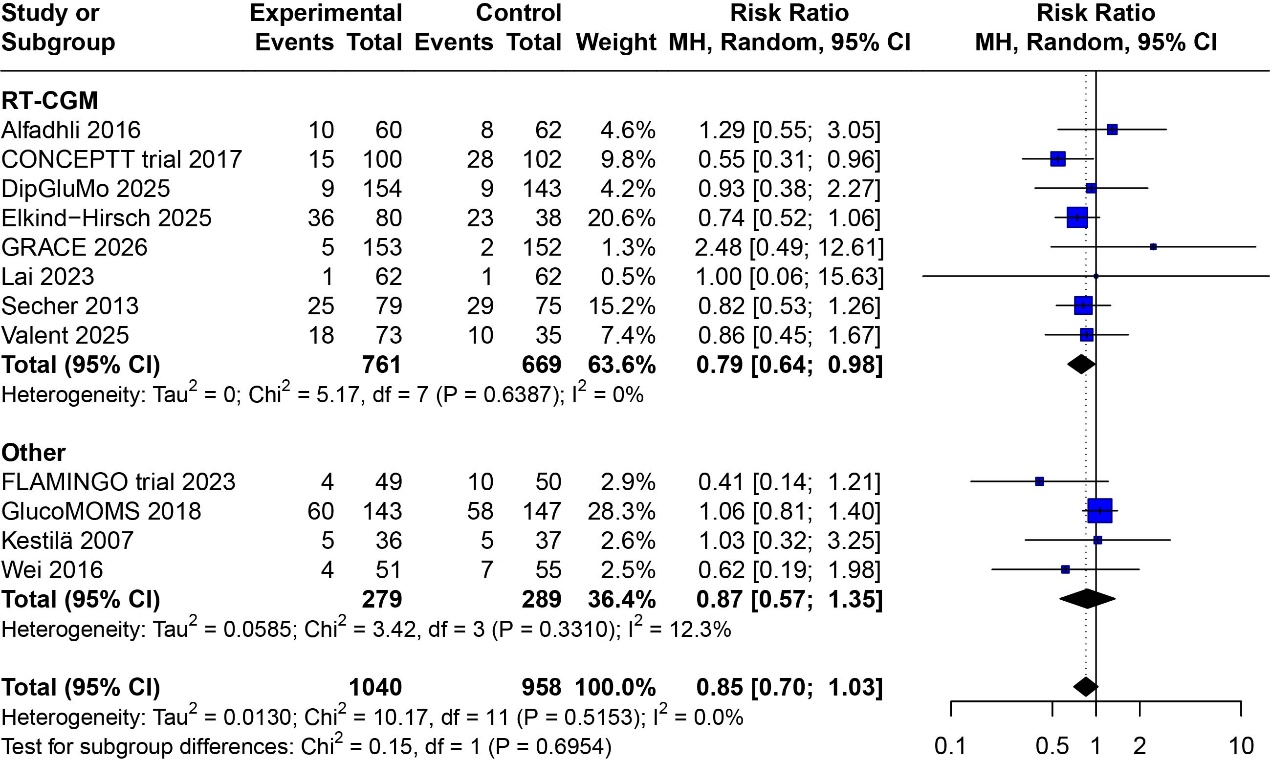


Figure 9: Forest plot for subgroup analysis for neonatal hypoglycemia, real-time CGM versus other CGM


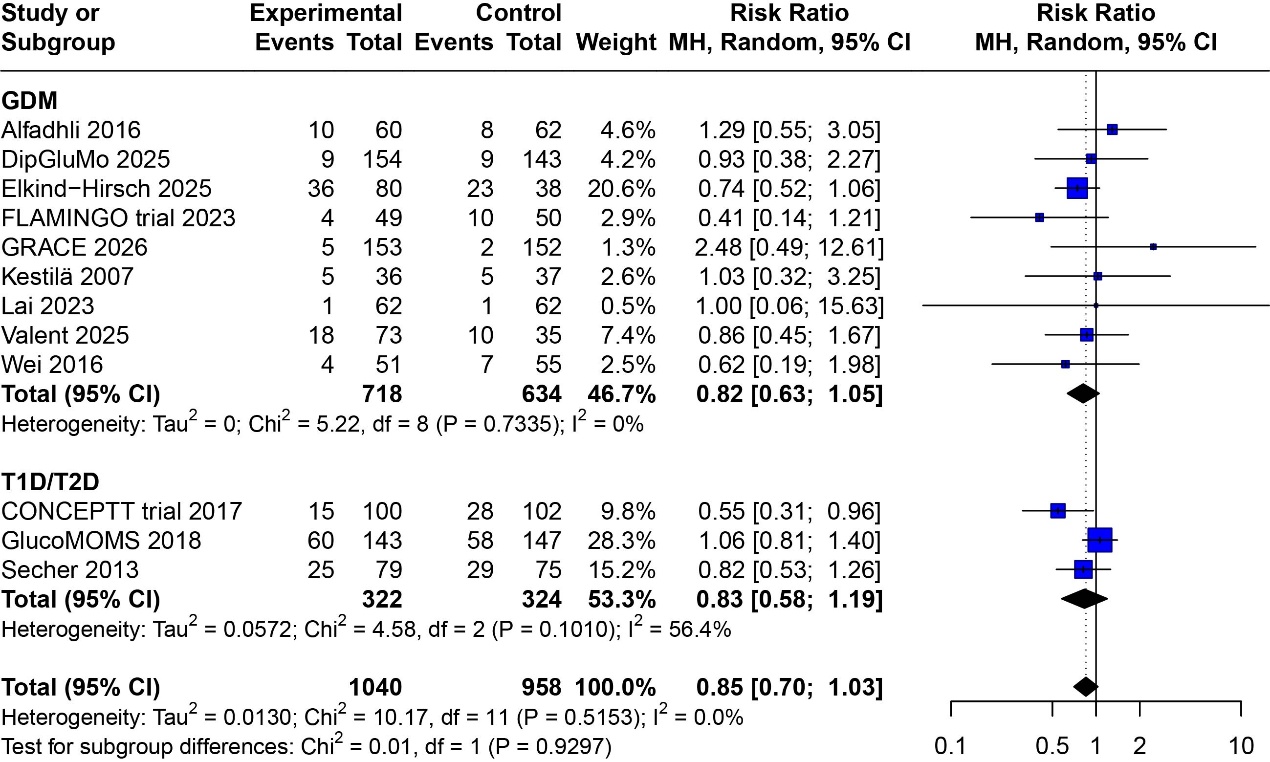


Figure 10: Forest plot for subgroup analysis for neonatal hypoglycemia, T1D/T2D versus GDM


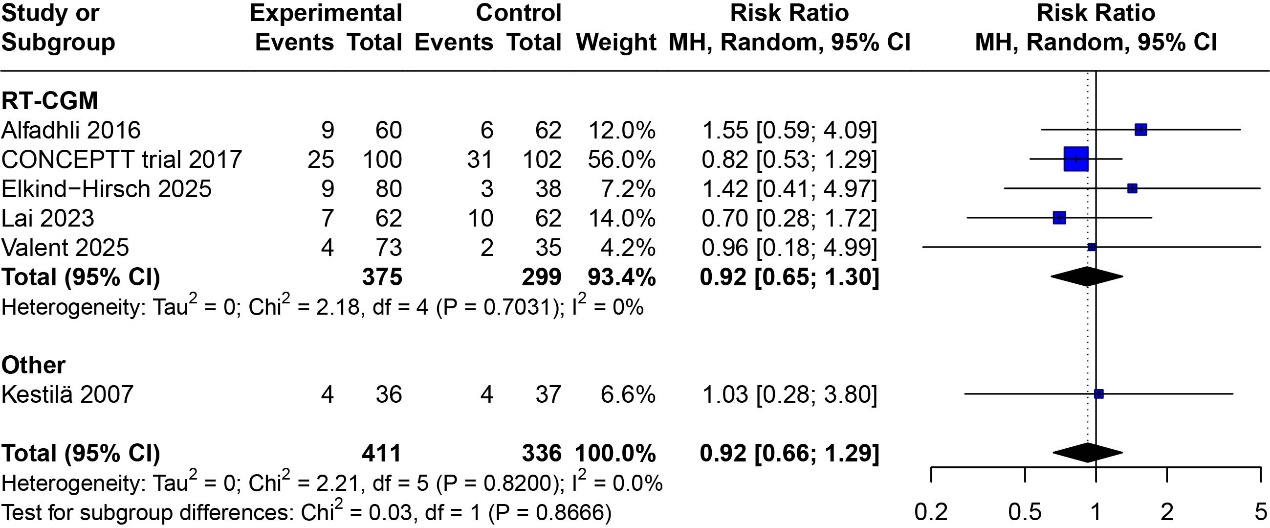


Figure 11: Forest plot for subgroup analysis for neonatal hyperbilirubinemia, real-time CGM versus other CGM


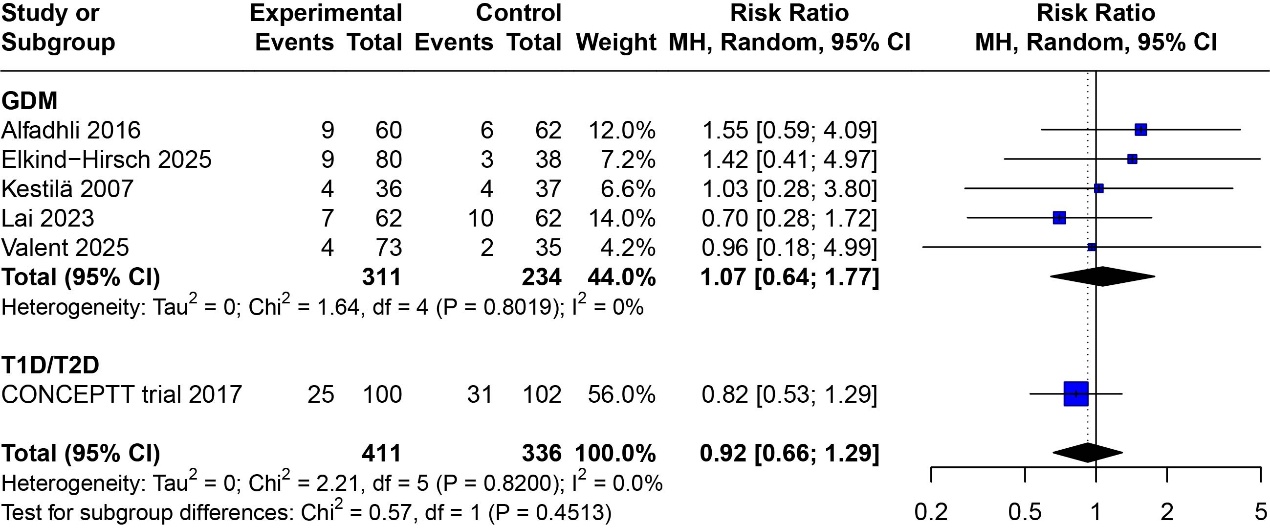


Figure 12: Forest plot for subgroup analysis for neonatal hyperbilirubinemia, T1D/T2D versus GDM


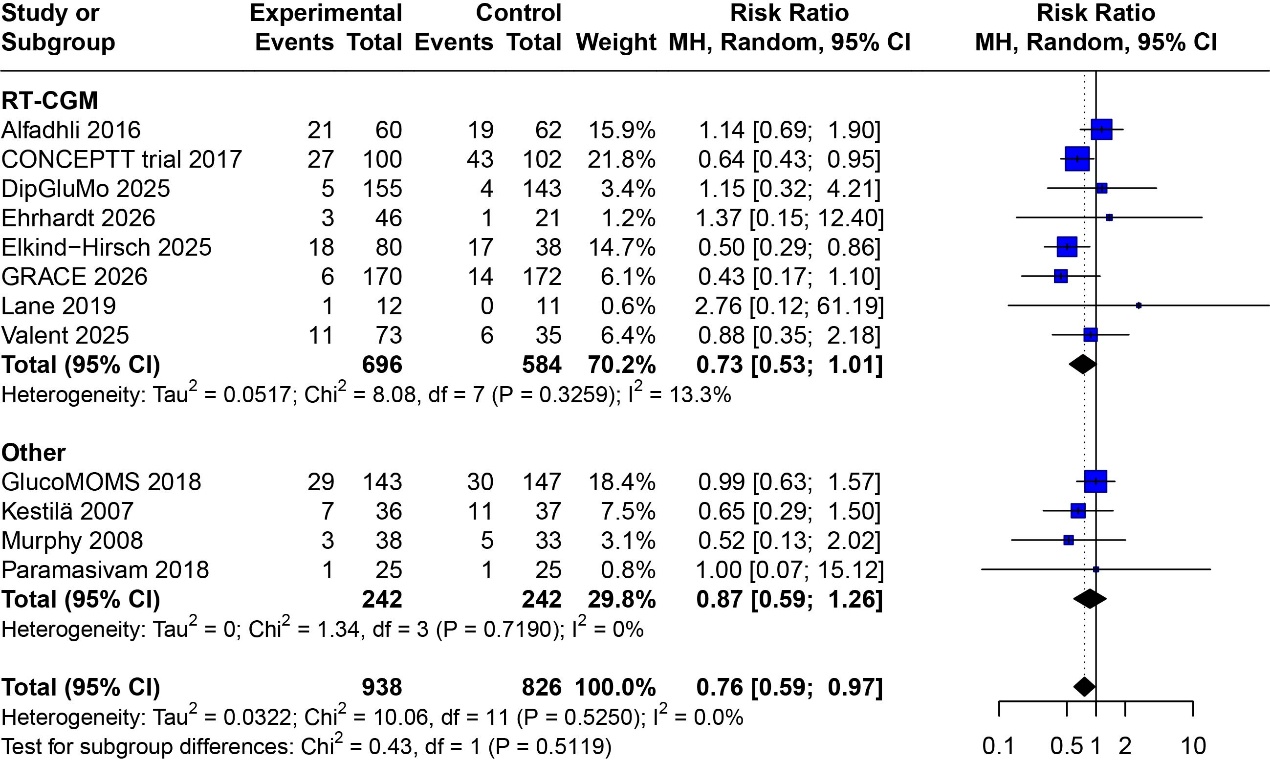


Figure 13: Forest plot for subgroup analysis for admission to the neonatal intensive care unit, real-time CGM versus other CGM


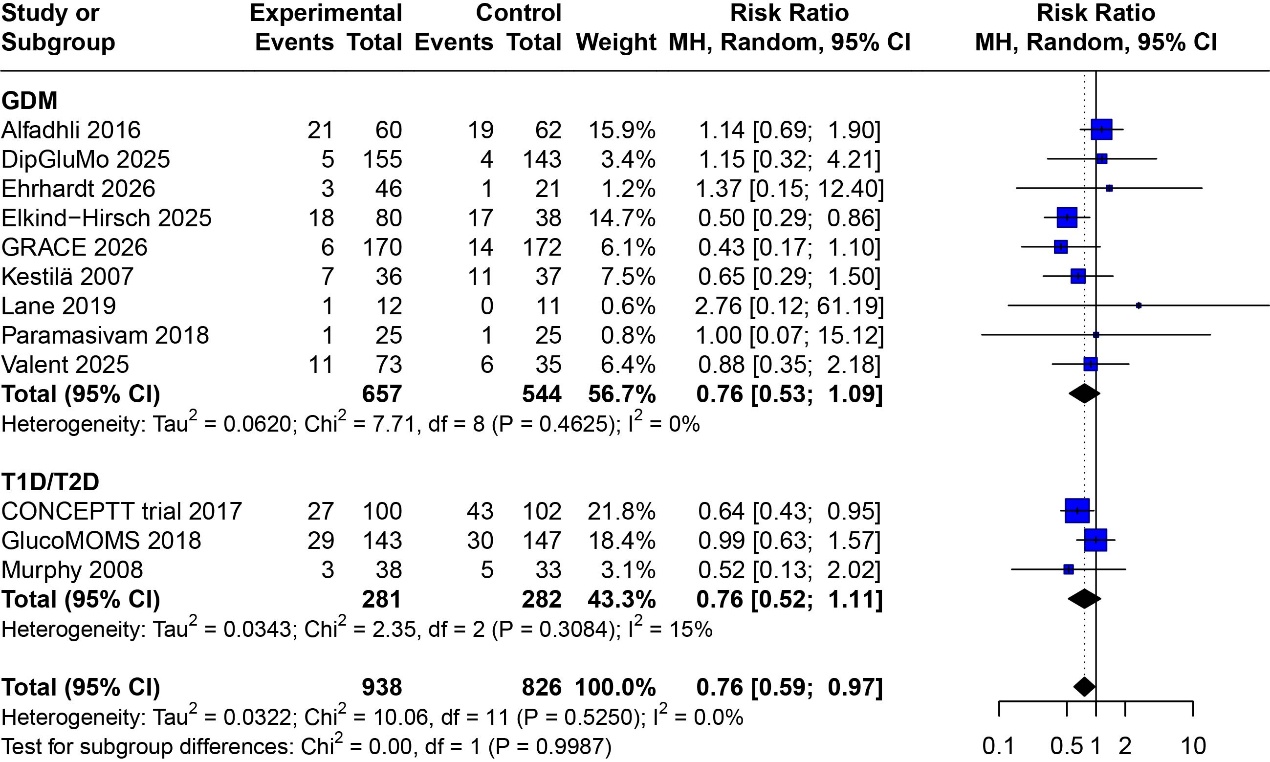


Figure 14: Forest plot for subgroup analysis for admission to the neonatal intensive care unit, T1D/T2D versus GDM
